# Application of optogenetic glial cells to neuron–glial communication

**DOI:** 10.3389/fncel.2023.1249043

**Published:** 2023-10-05

**Authors:** Sujin Hyung, Ji-Hye Park, Kyuhwan Jung

**Affiliations:** ^1^Precision Medicine Research Institute, Samsung Medical Center, Seoul, Republic of Korea; ^2^Division of Hematology-Oncology, Department of Medicine, Sungkyunkwan University, Samsung Medical Center, Seoul, Republic of Korea; ^3^Graduate School of Cancer Science and Policy, Cancer Biomedical Science, National Cancer Center, Goyang-si, Gyeonggi-do, Republic of Korea; ^4^DAWINBIO Inc., Hanam-si, Gyeonggi-do, Republic of Korea

**Keywords:** optogenetics, astrocyte, microglia, oligodendrocyte, Schwann cell, neuron–glia crosstalk

## Abstract

Optogenetic techniques combine optics and genetics to enable cell-specific targeting and precise spatiotemporal control of excitable cells, and they are increasingly being employed. One of the most significant advantages of the optogenetic approach is that it allows for the modulation of nearby cells or circuits with millisecond precision, enabling researchers to gain a better understanding of the complex nervous system. Furthermore, optogenetic neuron activation permits the regulation of information processing in the brain, including synaptic activity and transmission, and also promotes nerve structure development. However, the optimal conditions remain unclear, and further research is required to identify the types of cells that can most effectively and precisely control nerve function. Recent studies have described optogenetic glial manipulation for coordinating the reciprocal communication between neurons and glia. Optogenetically stimulated glial cells can modulate information processing in the central nervous system and provide structural support for nerve fibers in the peripheral nervous system. These advances promote the effective use of optogenetics, although further experiments are needed. This review describes the critical role of glial cells in the nervous system and reviews the optogenetic applications of several types of glial cells, as well as their significance in neuron–glia interactions. Together, it briefly discusses the therapeutic potential and feasibility of optogenetics.

## Introduction

Optogenetics is an innovative technology for controlling the complex nervous system, allowing for two-way interactions between neurons and glial cells. Numerous studies have shown that optogenetic treatment of neuronal cells promotes cell excitability (Adamantidis et al., 2007; Carter et al., 2010; Park et al., 2015; Hyung et al., 2019) and synaptic response (Lindsay et al., 2011; Mahn et al., 2016; Sinnen et al., 2017). These phenomena are followed by the firing of action potentials, thereby supporting the maintenance of nervous system health. Thus, optogenetics shows great promise for application in neuroscience research. But, studies on neuronal cells have reported limitations in controlling neuron–glia crosstalk and repairing injured nerves. Indeed, neuronal cells cannot spontaneously regenerate without the support of glial cells, which transform them into dedifferentiated phenotypes to promote nerve damage repair. As the importance of glial cells in neuroscience research has increased, advanced optogenetic tools targeting glial cells are needed to overcome the difficulty of regeneration.

Glial cells in the central nervous system (CNS) and peripheral nervous system (PNS) are known to play essential roles as structural and metabolic components of the complex nervous system. In particular, glial cells have been shown to play roles in the regulation of synaptic formation and activation, the propagation of action potentials for physical responses, and the secretion of neurotrophic factors for trophic support (Jessen and Mirsky, 2005; Zuchero and Barres, 2015). Recently, neuroscientists have predicted that optogenetics of glial cells might contribute to structural and functional maturation of the nervous system (Perea et al., 2014; Pelluru et al., 2016; Jung et al., 2019), which holds promise for improving nervous system function. Although glial cells lack the membrane components required to fire an action potential, they respond well to ion flux via voltage-sensitive ion channels and neurotransmitter receptors. Importantly, Ca^2+^ in optogenetically stimulated glial cells plays a critical role in modulating and developing the neural network, synaptic activity, and plasticity (Sasaki et al., 2012; Perea et al., 2014; Jung et al., 2019; Balachandar et al., 2020). Increasing evidence supports optogenetic stimulation of glial cells to enhance neuronal maturation and physiology, but the initial findings require verification, and further exploration using diverse experimental designs is needed.

## Optogenetics

Optogenetics is an innovative technology that integrates genetic and optical methods to precisely control the activity of excitable neurons using light. Through optogenetic manipulation, neurons can be genetically modified so that effector activity is induced by light. The principle of the optogenetic method is that a genetic component that responds to light, called opsin, is inserted into a neuron or a specific region of the brain, and related activities can be turned on or off using light (Deisseroth, 2010, 2011). One of the most widely used opsins is channelrhodopsin-2 (ChR2), which permits transmembrane movement of ions in and out of light-sensitive cells. This method can be used to depolarize neurons, resulting in precisely timed action potentials that propagate like electrical signals along the axon. Conversely, halorhodopsin (NpHR) is known as an inhibitory opsin and functions as a light-sensitive inward chloride pump, causing hyperpolarization of neurons. Using an opsin, researchers can easily manipulate the activity of a specific network, thereby elucidating cell–cell interactions in highly complex neural circuits. This is particularly useful for controlling one type of cell among numerous brain cells, and because it allows for photoactivation of biological processes at the millisecond timescale. Compared to electrical stimulation, which is insufficient for discriminating among nearby cells, optogenetics provides an unprecedented level of spatial and temporal resolution, making it advantageous for the precise control of neuronal activation.

Numerous studies have shown that optogenetics could be used to modulate cellular and molecular signaling pathways in real time with high precision (Boyden et al., 2005; Li et al., 2005; Bi et al., 2006; Stirman et al., 2011). In particular, this method has been investigated in the context of neurobiology, ophthalmology (Busskamp et al., 2012; Wu et al., 2013), and cardiac systems (Arrenberg et al., 2010; Bruegmann et al., 2010), and it has shown promise for improving organ dysfunction. Optogenetic control of well-defined biological events has allowed the monitoring of the complicated functions of nerve structures in living tissues. Optogenetics has provided new insight into the influence of individual cells on neighboring ones and on other cell types in a given region, which could help clarify the bidirectionality of communication between neurons and between neurons and glia. However, a deeper understanding of how optogenetic activation of a specific cell contributes to the control of neuronal activities in a wider region is still needed. Moreover, the best strategies for modulating neural circuits remain to be identified.

## Optogenetics in neurons

The importance of optogenetics has been in the spotlight for understanding how neurons (Adamantidis et al., 2007; Carter et al., 2010), neural circuits (Stuber et al., 2012; Sparta et al., 2013), and the nervous system (Alilain et al., 2008; Lindsay et al., 2011) operate under both normal and pathological conditions. In contrast to other techniques, optogenetics offers both high temporal precision and cell-type specificity. As a result, targeted neurons can be excited or inhibited rapidly, leading to the stimulation of neighboring cells and other neurons. To date, most optogenetic experiments have focused on direct neuronal excitability and inhibition, as well as their effects on neuromodulation (Stuber et al., 2012; Land et al., 2014), synaptic responses (Mahn et al., 2016; Sinnen et al., 2017), and behavior (Caggiano et al., 2014; Iyer et al., 2014). These studies have demonstrated that optogenetics is a powerful tool for behavioral analysis. Extensive optogenetics research has been conducted both *in vivo* and *in vitro*. *In vitro* studies have mainly investigated connectivity among individual cells, changes in cellular processes (Park et al., 2015; Lee et al., 2016), and neurotrophic factors secreted at the cellular level (Cheng et al., 2014; Park et al., 2015). *In vivo* research has assessed changes in behavior achieved through the excitation or inhibition of target neurons with an optical fiber connected to a laser or light-emitting diode (LED) implanted in freely moving animals (Towne et al., 2013; Bonin et al., 2016). This method enables the study of functional connectivity at multiple scales, from the neuron to the entire brain level, under physiological or pathological conditions.

In recent years, optogenetics has been increasingly used to explore the mechanisms underlying dysfunction caused by neurological disorders and injuries (Krook-Magnuson et al., 2013; Xie et al., 2013; Cheng et al., 2014; Lim et al., 2014). Using an optogenetics approach, Lim et al. (2014) studied neural network changes in both cortical hemispheres after a cortical stroke and reported heterogeneous recovery of the injured region, even in the intact hemisphere, as reflected in changes in functional connections. These results are consistent with the view that stroke affects networks throughout the brain and supports the involvement of intact regions in stroke recovery. In addition, optogenetics has been used to investigate cell types associated with cortical excitability and recovery. Xie et al. (2013) assessed responses to optogenetic stimulation of cortical neurons and electrical stimulation of the forelimb. Electroencephalography and electromyography were used to examine sensory processing after global ischemia, and the acute effects of global ischemia were also investigated using optogenetics. Optogenetics has also been applied to neurological disorders in studies investigating the role of GABAergic cells in the hippocampus in temporal lobe epilepsy (Krook-Magnuson et al., 2013). Optogenetic interventions involving the stimulation of parvalbumin (PV)-expressing GABAergic neurons with ChR2 were found to attenuate electrical seizures and reduce the frequency of behavioral seizures. These results demonstrate the potential application of optogenetics in therapeutic interventions to control spontaneous seizures in cases of temporal lobe epilepsy.

Furthermore, optogenetics has been widely used in pre-clinical studies as a rehabilitative and therapeutic tool for neurodegenerative diseases (Gradinaru et al., 2009; Yoon et al., 2014; Seeger-Armbruster et al., 2015). In the ventroanterior motor thalamus, glutamatergic neurons can be activated through optogenetics to modulate movement in acute drug-induced Parkinsonian rats. An optogenetic stimulation pattern that mimicked real neuronal activity or theta-burst patterns showed significantly improved effectiveness (Seeger-Armbruster et al., 2015). Moreover, optogenetic inhibition of the subthalamic nucleus improved the motor symptoms of Parkinson’s, including forelimb akinesia, by reducing the firing of inhibitory neurons in areas such as the internal globus pallidus and substantia nigra pars reticulata (Yoon et al., 2014). Optogenetics has also been used to study Alzheimer’s disease (Yamamoto et al., 2015; Etter et al., 2019). For example, Etter et al. (2019) reported that optogenetic activation of PV neurons restored slow gamma oscillations in the hippocampus and rescued spatial memory in a mouse model of Alzheimer’s disease. It has also been reported that optogenetic stimulation of dentate gyrus neurons reduced memory impairment and promoted the reactivation of previously activated neural ensembles during memory encoding (Tonegawa et al., 2015). These results suggest that optogenetic stimulation could serve as an innovative treatment for neurodegenerative diseases.

Several optogenetic studies have focused both on individual neurons (Park et al., 2015) and their interactions with surrounding cells, especially glial cells, which promote myelination (Gibson et al., 2014; Lee et al., 2016; Hyung et al., 2019). The myelin sheath is an electrical insulator formed by oligodendrocytes in the CNS and Schwann cells in the PNS. It wraps around axons to provide electrical insulation, enveloping and protecting the axons while also increasing the propagation speed of the action potential and providing neurotrophic factors to the axons. When the myelin sheath is damaged, signal conduction of the affected nerve is impaired, which can affect sensation, movement, cognition, and other functions depending on the nerve involved; in severe cases, this can lead to demyelinating diseases such as multiple sclerosis (Genain et al., 1999; Zhou et al., 2006; Fancy et al., 2010), Charcot–Marie–Tooth disease (Patel et al., 1992; Sereda et al., 2003; Berger et al., 2006), and Guillain−Barré syndrome (Koski, 1990; Khalili-Shirazi et al., 1993; Ruts et al., 2012). Reforming a myelin sheath following injury is difficult; numerous methods have been investigated, including use of neurotrophic factors (McTigue et al., 1998; Chan et al., 2001; Gingras et al., 2008), stem cells (Gupta et al., 2012; Uchida et al., 2012), autologous (den Dunnen and Meek, 2001; Zhang et al., 2010) or artificial nerve grafts (Xu et al., 2003; Huang et al., 2012), and electrical stimulation (Brushart et al., 2002; Ishibashi et al., 2006; Huang et al., 2010). However, their therapeutic efficiency has been limited by indiscriminate stimulation of the injured area. Therefore, optogenetic techniques are increasingly being used to promote myelination, and have shown remarkable efficacy. Several studies have reported that the number of oligodendrocytes and myelin thickness in the CNS increased with optogenetic stimulation, both *in vivo* and *in vitro* (Gibson et al., 2014; Lee et al., 2016; Yamazaki et al., 2019). For example, Gibson et al. (2014) demonstrated that optogenetic stimulation of neurons in the premotor cortex induced the proliferation of oligodendrocyte precursor cells (OPCs), some of which differentiated into mature oligodendrocytes and increased myelin thickness in active neuronal circuits. In addition, using a three-dimensional microfluidic chip, Hyung et al. (2019) demonstrated that optogenetic stimulation of motor neurons promoted the differentiation of Schwann cells and enhanced axon outgrowth. Moreover, they showed that the thickness of the myelin sheath was similar to that under normal conditions.

## Optogenetics in glia

### Astrocytes

Astrocytes are the most abundant non-neuronal cells in the brain, composing approximately 50% of the volume of the adult mammalian brain. For a century, studies considered astrocytes merely as structural supporters for neuronal cells. Astrocytes typically extend their processes, tightly contacting the synapses and blood vessels in their surroundings, positioning them well to maintain the structural integrity of the blood–brain barrier (Abbott, 2002; Cabezas et al., 2014). They express transporters on their membranes for ions, enabling them to maintain brain extracellular homeostasis by regulating extracellular K^+^ and pH levels (Walz, 2000; Perea et al., 2014). Furthermore, they express glutamate transporters, allowing them to clear away excess released glutamate (Rothstein et al., 1994; Sutherland et al., 1996; Danbolt, 2001; Huang and Bergles, 2004; Tzingounis and Wadiche, 2007).

During the last few decades, studies have indisputably demonstrating that astrocytes not only support and maintain the neuronal cells but also contribute significantly to the regulation of synaptic transmission. Astrocytes express many neurotransmitter receptors, including metabotropic (mGluR) and ionotropic (AMPAR) glutamate receptors, ATP receptors, and GABA receptors (Porter and McCarthy, 1997; Hamilton and Attwell, 2010; Kimelberg and Nedergaard, 2010; Allen, 2014). These receptors indicate that not only neurons but also astrocytes can be activated by the neurotransmitters released from the presynaptic terminal. Supporting studies have shown that the activation of synaptic transmission induces intracellular calcium increases in the astrocytes (Wang et al., 2009; Bazargani and Attwell, 2016). Furthermore, it is established that astrocytes can release some neurotransmitters such as glutamate, D-serine, ATP, and GABA, collectively referred to as “gliotransmitters” (Parpura et al., 1994; Mothet et al., 2000; Yoon and Lee, 2014). The release of these gliotransmitters often occurs when G protein-coupled receptors (GPCR) are activated, leading to sequential downstream signaling pathways like phospholipase C, adenylate cyclase, and inositol 1,4,5-trisphosphate (IP3), which can cause an increase in intracellular calcium in astrocytes (Sheppard et al., 1997; Agulhon et al., 2008; Petravicz et al., 2008). Since they are connected via gap junctions composed of connexin channels (Theis et al., 2003), which allow cAMP and calcium to move around (Harris, 2007; Orthmann-Murphy et al., 2008), the intracellular calcium increase in a single astrocyte can be passed to other astrocytes, initiating the calcium waves (Hofer et al., 2002; Theis et al., 2003; Scemes and Giaume, 2006; Theis and Giaume, 2012). Increased intracellular calcium in astrocytes is considered one of the common factors that drive the release of gliotransmitter (Allen, 2014; Bazargani and Attwell, 2016). Gliotransmitters released from astrocytes are able to facilitate the excitability of neighboring neurons (Perea et al., 2009; Araque and Navarrete, 2010; Allen, 2014; Sahlender et al., 2014). The consequences depend on the location and the activated receptors of the neurons (Araque et al., 2001; Bezzi and Volterra, 2001; Liu et al., 2004; Perea et al., 2009; Sasaki et al., 2011). Based on these evidences of interaction and communication between astrocytes and neurons, a concept of the “tripartite synapse” has been proposed (Agulhon et al., 2008).

As mentioned previously, astrocytes were for a long time known to be non-excitable cells and therefore were assumed not to be influenced by membrane potential or by neurotransmitters. However, with the development of optogenetics, many studies have shown that astrocytes can truly be a practical tool to investigate the role of astrocytes in deeper aspects of higher brain functions such as respiration, depression, sleep, memory, disease, pain, and much more to be investigated in the near future (Li et al., 2012; Berlinguer-Palmini et al., 2014; Figueiredo et al., 2014; Ono et al., 2014; Bang et al., 2016).

**Figure 1:**
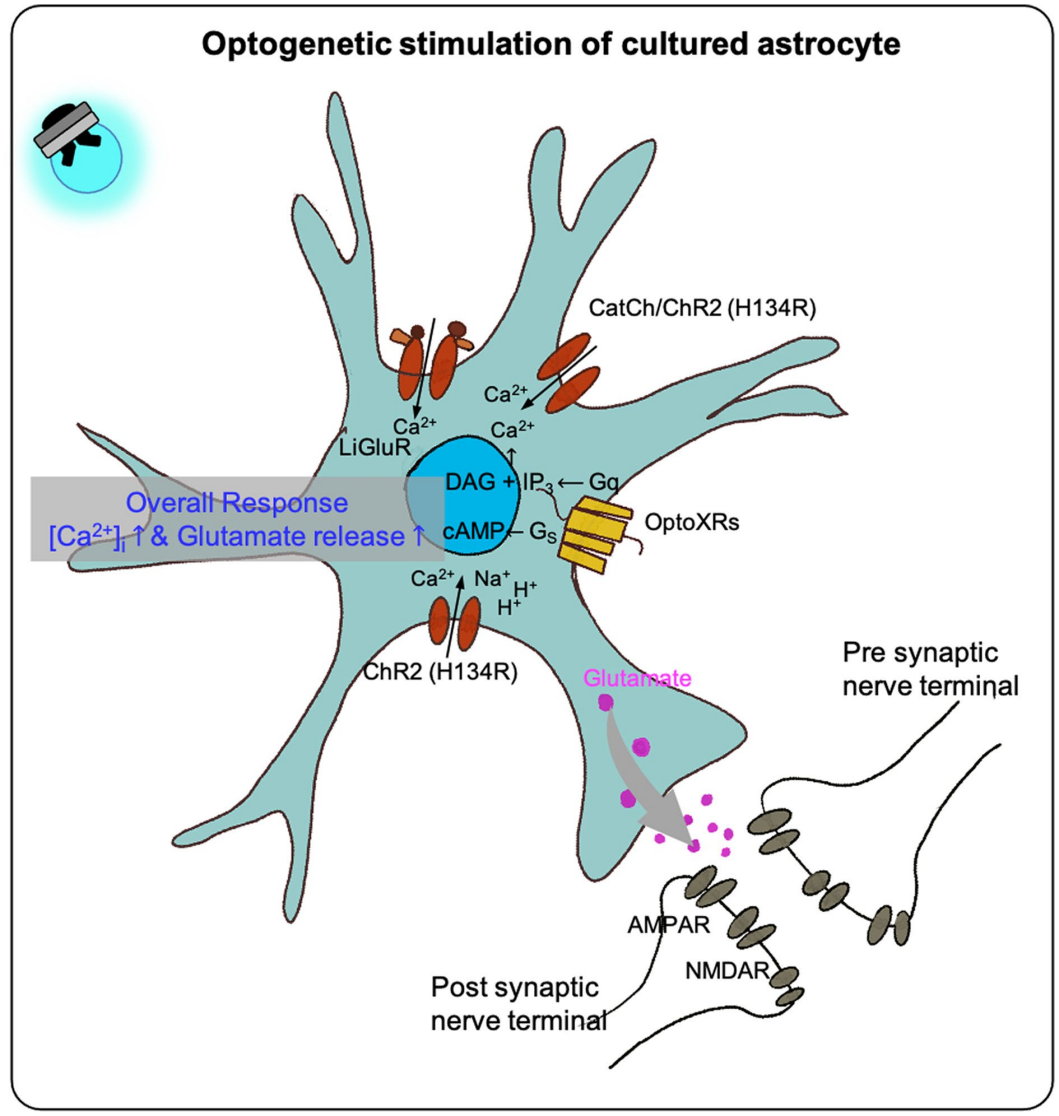
The effect of optogenetic stimulation on cultured astrocytes. Previous studies shown that the optogenetic stimulation on the cultured astrocytes transfected with different optogenetic effectors (LiGluR, CatCh, ChR and its variants, OptoXR) showed increased intracellular calcium concentration in overall, some resulted in glutamate release (see Table 1).

In optogenetic manipulation studies involving astrocytes, both primary astrocytes culture and immortal cell lines have demonstrated that transfected opsins can respond to photostimulation, leading to intracellular calcium increases and gliotransmitter release *in vitro* (Figure 1; Table 1).

**Table 1:** Optogenetic stimulation of cultured astrocytes.

References	Description
Li et al. (2012)	Shown that photostimulation of light-gated calcium permeable ionotropic GluR6 glutamate receptor (LiGluR)-expressing astrocytes elicit calcium transient in cultured cortical astrocytes which led to glutamate-mediated signaling in LiGluR-positive cells and neighboring LiGluR-negative cells.
Berlinguer-Palmini et al. (2014)	Shown photostimulation of ChR2 expressing astrocytes elicit intracellular calcium increase and electrophysiological responses and also in the co-cultured astrocytes and neurons which do not express ChR2. NMDA and AMPA receptor antagonists suppressed the calcium response of Ch2 negative cells which showed their calcium response was due to glutamate release from the Ch2 positive astrocytes.
Ono et al. (2014)	Shown photostimulation of ChR2-expressing astrocytes exhibited intracellular sodium and calcium increase, acidification, glutamate release, reduced proliferation. Short period photostimulation for minutes elicited calcium transients in the co-cultured ChR2-negative neurons and long period photostimulation for several days lead to apoptosis of co-cultured ChR2-negative neurons. AMPA receptor antagonist blocked both responses, demonstrating astrocytes activation lead to glutamate release which provokes intracellular calcium increase and cytotoxic cell death.
Figueiredo et al. (2014)	Developed an array of adenoviral vectors (AVV) with ChR2-like actuators, including an enhanced ChR2 mutant (H134R), and a mutant with improved calcium permeability (Ca^2+^translocatingchannelrhodopsin, CatCh), showing that intracellular calcium elevations evoked by ChR2(H134R) and CatCh in astrocytes are largely due to release of calcium from the intracellular stores. The autocrine action of ATP which is released under these conditions and acts on the P2Y receptors also contributes to the intracellular calcium elevations. Activation of light-sensitive G-protein coupled receptors (opto-adrenoceptors) like optoα1AR (Gq-coupled) and optoβ2AR (Gs-coupled) resulted in astrocytic intracellular calcium increases which were suppressed by blocking the corresponding intracellular signaling cascade (phospholipaseC and adenylate cyclase, respectively). the bulk of intracellular calcium responses evoked using either optoAR was blocked by an ATP degrading enzyme, apyrase, or a P2Y1 receptor blocker, MRS 2179, indicating that they are to a large extent triggered by the autocrine action of ATP.

**Figure 2:**
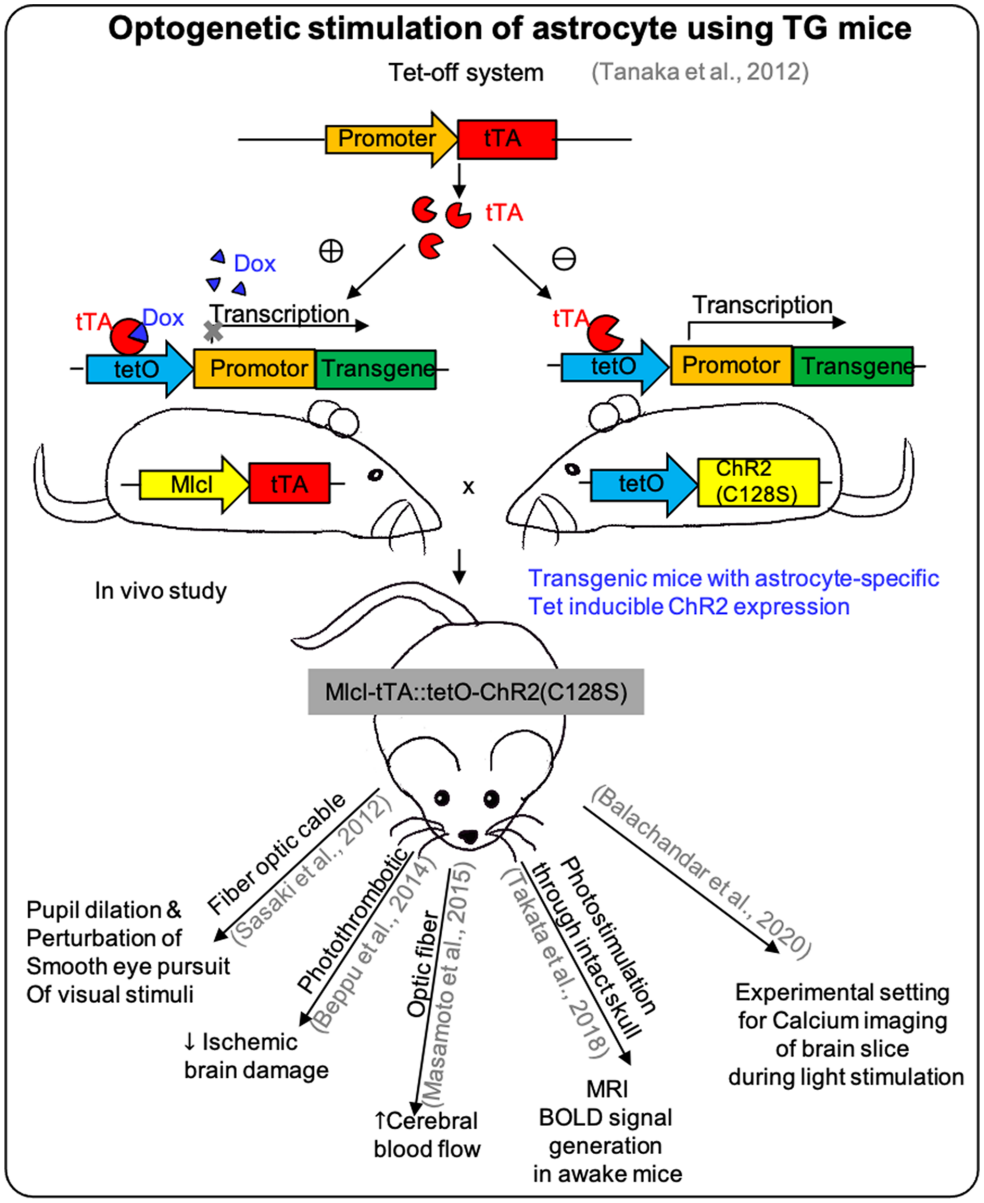
The effect of optogenetic stimulation on astrocytes using TG mice. Researches used the Tet-off system on TG mice for expressing ChR2 specifically on astrocytes to demonstrate the *in vivo* effect of optogenetic stimulation and showed different responses depending on the site of effectors (see Table 2).

Two main approaches have been employed for the *in vivo* expression of astrocytes-specific opsins (Figure 2).

One is to use the astrocytes-specific opsin transgenic or knock-in mice (Table 2). Both Cre/loxP and tetO-tTA systems can be used for astrocytes-specific opsin-transgenic mice, which can be generated by mating astrocytes-specific Cre or tTA mice with floxed-stop-opsin knock-in mice or tetO-opsin transgenic mice (Cho et al., 2016).

**Table 2:** Optogenetic stimulation of astrocytes using transgenic mice.

References	Description
Tanaka et al. (2012)	Generated astrocyte specific ChR2 transgenic mice by knocking in a transgene cassette encoding tetO-driven ChR2(C128S) downstream of housekeeping beta-actin gene and crossed to tTA driver lines with astrocyte-specific promotor Mlc1 to express sufficient amounts of ChR2 in astrocytes for optogenetic astrocyte manipulation.
Sasaki et al. (2012)	With the double-transgenic mice containing Mlc1-driven tTA and tetO driven ChR2(C128S) (Tanaka et al., 2012), demonstrated that photostimulation of ChR2 on cerebellar astrocytes Bergmann glia (BG) can trigger electrophysiological responses from the acute brain slices of the TG mice and glutamate release, firing nearby Purkinje cells (PCs) resulting long-term plasticity between parallel fibers and PCs. Photostimulation using fiber-optic cable installed above skull without injury induced reactive gliosis was sufficient to induce c-fos (surrogate marker for cellular activation) from ChR2 expressing Bergmann glia (BG, a specialized subtype of astrocytes in the cerebellum). *In vivo* photostimulation with fiber-optic cable inserted into the cerebellar flocculus caused pupil dilation and perturbation of smooth eye pursuit of visual stimuli in head-fixed mice.
Beppu et al. (2014)	With the same mouse line (Tanaka et al., 2012), shown stimulation of astrocytic ChR2 leads to intracellular acidification and release of glutamate, followed by inward excitatory current in surrounding PCs which could be blocked by glutamate receptor blocker and transporter blocker cocktail and non-competitive AMPA and kainite receptor antagonist confirming glutamate involvement in signaling between BG and PC. Whereas activation of ArchT, a light-gated outward proton pump led to intracellular alkalization and blocks glutamate release, which limits ischemic brain damage. *In vivo* photostimulation of ArchT-expressing BG reduced ischemic brain damage.
Masamoto et al. (2015)	With the same mouse line (Tanaka et al., 2012), shown photostimulation of cortical astrocytes expressing ChR2 induced a fast transient increase in cerebral blood flow via K^+^ signaling to the vascular smooth muscle cells.
Takata et al. (2018)	With the same mouse line (Tanaka et al., 2012), shown causal relationship between light stimulated ChR2 expressing astrocyte activity and blood oxygenation level-dependent (BOLD) signal generation using of MRI in awake transgenic mice. Activation of astrocytes, augmented synthesis of acetyl-carnitine (AC) from glucose, which consumed oxygen.
Balachandar et al. (2020)	Generated experimental setting that uses live adult murine brain slices (2–5 months) from a knock-in model expressing ChR2(C128S) in cortical astrocytes, loaded with Rhod-2 AM to elicit robust calcium response to light stimulation. Developed and standardized a protocol for brain extraction, sectioning, Rhod-2 AM loading, maintenance of slice health, and Ca^2+^ imaging during light stimulation which successfully applied to optogenetically control adult cortical astrocytes, which exhibit synchronous patterns of calcium activity upon light stimulation, drastically different from resting spontaneous activity.

**Figure 3:**
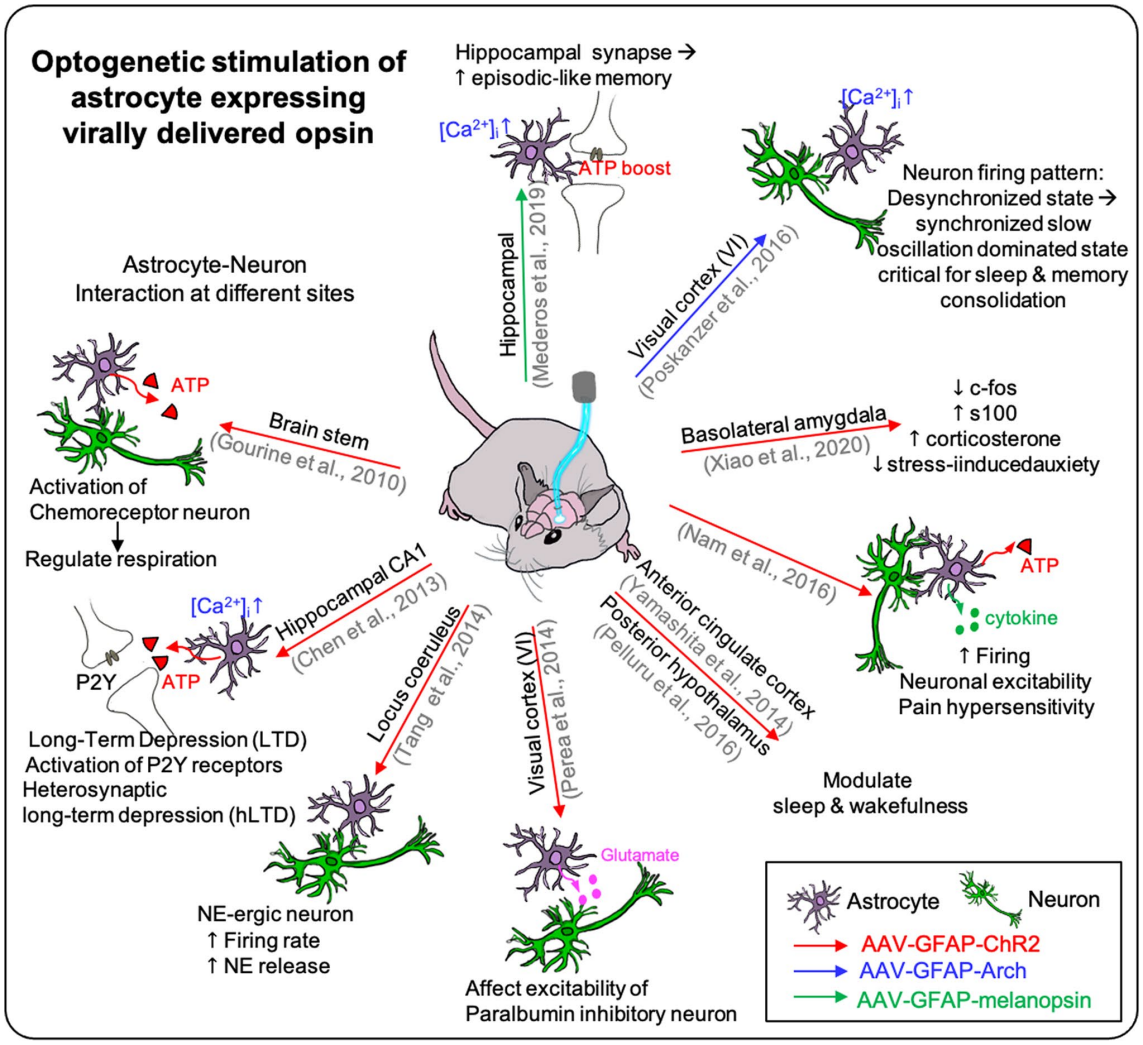
The effect of optogenetic stimulation on astrocyte expressing virally delivered opsin. Virally delivered opsins are used on many different sites on the brain in the previous studies. It showed the effect of optogenetic stimulation on the opsin-expressing astrocyte itself and showed the effect on the neighboring neurons affected by the photostimulation of the astrocytes. Furthermore, it demonstrated the optogenetic stimulation could affect higher brain function such as respiration, depression, sleep, pain, and memory (see Table 3).

The other approach is to use recombinant adeno-associated virus (AAV) for opsin construct expression (Figure 3).

For example, viral vectors encoding opsin genes under the astrocyte-specific promoter (e.g., GFAP) are used to express astrocyte-specific ChR2 in the mouse brain (Table 3).

**Table 3:** Optogenetic stimulation of astrocytes expressing virally delivered opsins.

References	Description
Gradinaru et al. (2009)	Used anesthetized parkinsonian rodent model with 6-hydroxydopamine (6-OHDA) injected into the right medial forebrain bundle unilaterally causing a loss of nigral dopaminergic cells to examine whether local astrocytes contribute to the therapeutic effect of deep-brain stimulation (DBS) delivered to the subthalamic nucleus (STN) to relieve tremor in Parkinson’s disease. Photostimulation to ChR2-expressing astrocytes in the STN reversibly inhibited firing of STN neurons in 6-OHDA-treated animals but did not change pathological motor behavior in parkinsonian rats.
Gourine et al. (2010)	Shown astrocytes residing near the ventral surface of the medulla oblongata (*VS*) showed increased intracellular calcium in response to pH decrease in anesthetized rats. And with brainstem slices, decrease in pH elicited sustained ATR release in the *VS* region and *VS* astrocyte extracellular ATP-dependent calcium response. To mimic this, AAV encoding enhanced GFAP promotor-driven ChR2(H134R) was injected to brainstem. Photostimulation induced calcium transient in ChR2-expressing astrocytes and extracellular ATP-dependent long lasting depolarization in neighboring chemo-sensitive neurons in the retrotrapezoid nucleus (RTN). Following *in vivo* photostimulation, ATP released from activated astrocytes in the brainstem and activated chemoreceptor neurons and regulated respiration.
Chen et al. (2013)	Shown photostimulation activated of AAV encoding GFAP promotor-driven ChR2 expressing astrocytes in the hippocampal CA1 region triggers the elevation of intracellular calcium and ATP release which result in long-term depression (LTD) of synapses on neighboring neurons and activation of P2Y receptors, mediating heterosynaptic long-term depression (hLTD) accompanying long-term potentiation (LTP) induction.
Tang et al. (2014)	Photostimulation activated AAV encoding GFAP promoter-driven ChR2(H134R)-expressing astrocytes, which released L-lactate. This L-lactate excited locus coeruleus (LC) norepinephrine (NE)-ergic neurons on the organotypically cultured brain slices, leading to an increased firing rate and delayed depolarization, which triggered the release of norepinephrine (NE). Optogenetic activation of either optoβ2AR or ChR2(H134R) could also trigger NE release.
Perea et al. (2014)	Shown *in vivo* stimulation of AAV encoding GFAP promotor-driven ChR2-expressing visual cortex (V1) astrocytes enhanced both excitatory and inhibitory synaptic transmission in parvalbumin expressing inhibitory neurons nearby. This regulatory effect of astrocytes was dependent on the metabotropic glutamate receptor (mGluR1), implicating glutamate as a gliotransmitter in the response upon optogenetic stimulation.
Yamashita et al. (2014)	Shown *in vivo* optogenetic astrocytes Ch2R activation in the anterior cingulate cortex during neuropathic pain can modulate wakefulness and sleep.
Pelluru et al. (2016)	Shown ChR2 stimulation of astrocytes in the posterior hypothalamus dramatically induced sleep during the active phase of the sleep–wake cycle affirming the involvement of astrocytes in sleep–wake regulation.
Poskanzer and Yuste (2016)	Generated astrocyte-specific expression opsin in the visual cortex (V1) by injecting AAV encoding Cre-dependent Arch in the transgenic mice expressing GFAP promotor-driven Cre. Photostimulation of Arch expressing astrocytes triggered calcium transient at the processes but not in the soma. Calcium response of neighboring Arch-negative cells were not affected. *In vivo* optogenetic stimulation of astrocytes in the V1 resulted in calcium transient and small increase in extracellular glutamate. And changed neuron firing pattern in V1 from a desynchronized state to synchronized slow oscillation-dominated state which is critical for sleep and memory consolidation.
Nam et al. (2016)	Shown *in vivo* stimulation of spinal cord AAV encoding GFAP promotor-driven ChR2(H134R) expressing astrocytes with an optogenetic fiber in rats induces mechanical and thermal pain via release of ATP and cytokines from astrocytes, which causes neuronal excitability and pain hypersensitivity.
Mederos et al. (2019)	Shown selective expression of AAV encoding GFAP104 (short version of GFAP) promotor-driven melanopsin, a G-protein-coupled photopigment in astrocytes trigger calcium signaling in astrocytes. Melanopsin stimulated robust IP3-dependent calcium signals in astrocytic fine processes and evoke an ATP/Adenosine-dependent transient boost of hippocampal excitatory synaptic transmission. Under low-frequency light stimulation conditions, melanopsin-transfected astrocytes can trigger long-term synaptic changes. *In vivo*, melanopsin-astrocyte activation enhances episodic-like memory.
Xiao et al. (2020)	Optogenetic activation of basolateral amygdala (BLA) glutamatergic neurons did not alleviate anxiety in stressed mice, but optogenetic activation of BLA astrocytes had a positive effect, relieving stress-induced anxiety. Optogenetic manipulation fully restored the unpredictable chronic mild stress (UCMS)-induced behavioral and neurobiological dysfunctions, including anxiety-like behavior, lower c-Fos expression in the BLA, S100 overexpression in the BLA, and higher serum corticosterone concentration.

Studies have been demonstrating clearly that the optogenetic manipulation can regulate astrocyte gliotransmitter release and subsequent synaptic transmission. Of course, there are certain limitations of current optogenetic astrocyte manipulation. For example, the intracellular calcium increase in astrocytes via optogenetic ChR2 activation does not exactly recapitulate physiological astrocyte calcium elevation, which is mostly due to GPCRs activation. Additionally, astrocytic calcium responses are distinct depending on which compartment of the cell is involved because different compartments have different expression of receptors and sources for calcium. In this regard, some review papers suggest the utilization and development of more optogenetic tools, such as optoXR (Airan et al., 2009) or OptoSTIM1 (Kyung et al., 2015). These tools include cell-signaling modifying opsins, such as chimeric opsins (the Gq-coupled optoa1AR and the Gs-coupled optobAR), which have been shown to induce calcium increases in cultured astrocytes as efficiently, if not more efficiently, as ChR2 (Figueiredo et al., 2014; Adamsky and Goshen, 2018).

While neuronal optogenetics allows for precise control of membrane potential and the timing of action potentials, astrocyte optogenetics involves influencing processes like neurotransmitter regulation, synaptic plasticity, and the ionic environment, which are critical for overall brain homeostasis. However, glial optogenetics may not achieve the same level of timing precision as neuronal optogenetics, since it often involves modulating slower processes such as calcium signaling in astrocytes. One notable finding (Figueiredo et al., 2014; Gerasimov et al., 2021) is related to the potentiation of field potentials during optogenetic activation of astrocytes expressing Opto-a1AR (metabotropic opsin). This potentiation is associated not only with the release of glutamate but also gliotransmitters like D-serine, crucial for long-term changes in plasticity. The release of D-serine occurs through calcium-dependent exocytosis and other pathways. Activation of Opto-a1AR significantly increases intracellular calcium concentrations, facilitating D-serine release from astrocytes. This differs from ChR2 activation, which does not produce a comparable increase in intracellular calcium.

The significance of astrocyte optogenetics in this context lies in its ability to influence neuronal networks in a nuanced and precise manner. Astrocytes are pivotal in modulating synaptic transmission, plasticity, and neurovascular coupling. Targeting astrocytes with optogenetics not only allows for the modulation of neurotransmitter release but also affects gliotransmitter dynamics like D-serine, critical for long-term plasticity. This approach holds promise for correcting network dysfunctions seen in neurodegenerative disorders.

However, it is essential to acknowledge the potential drawbacks of astrocyte stimulation, including the conversion of astrocytes into reactive glia with cytotoxic effects. This conversion can harm neurons and exacerbate neuroinflammation. *In vivo* experiments are necessary to validate the study’s parameters and refine the approach to mitigate potential negative effects on brain function. Even with some restrictions and limitations of the current optogenetic technique, it is truly undeniable that optogenetics is a valuable tool for elucidating the more physiological roles of astrocytes in higher-order brain functions.

### Microglia

Microglia are immune cells of central nervous system, composing 5–10% of total brain cells. They constantly survey the microenvironment in the brain and spinal cord through their ramified processes. Under normal healthy conditions, microglia sensitively observe their surrounding area with their long and thin branches, which spans about 50 μm in diameter. Ramified microglia in the resting state under normal physiological conditions are able to search for and identify immune threats while maintaining homeostasis in the CNS (Davis et al., 1994; Aloisi, 2001; Christensen et al., 2006). The resting potential of microglia is around -40 mV, maintained by THIK-1 (Tandem of P domains in a Weak Inward rectifying K^+^ channel 1) and potentially by TRP (transient receptor potential) and chloride-conducting channels (Newell and Schlichter, 2005; Madry et al., 2018). THIK-1 is identified as the primary potassium (K^+^) channel expressed in microglia, and it is a constitutively active, with its activity being enhanced by P2Y12 receptors. Inhibiting THIK-1 function through pharmacological means or gene knockout results in the depolarization of microglia, reducing their ramification, and thus impairing their surveillance capabilities. Interestingly, blocking P2Y12 receptors does not affect membrane potential, ramification, or surveillance. This suggests that THIK-1 plays a vital role in maintaining microglial membrane potential, which is crucial for immune surveillance and the release of pro-inflammatory cytokines like interleukin-1β from activated microglia. Besides, TRP channels are a family of ion channels that include both cation and anion channels. Some TRP channels can allow the flow of calcium ions (Ca^2+^) into microglia, which can influence membrane potential and cellular responses. These channels may be involved in detecting changes in the extracellular environment, such as injury or inflammation, and can contribute to microglial activation. Chloride channels are responsible for regulating the flow of chloride ions (Cl^−^) into or out of the cell membrane. The movement of chloride ions can affect the overall membrane potential of microglia. These channels play a role in maintaining the electrochemical balance within the cell, which is essential for normal cellular functions, including immune responses.

Microglia was previously classified as non-excitable cells, but they do exhibit strong electrophysiological stimulus–response features. Unlike neurons and astrocytes, microglia have no reported studies showing direct optogenetic microglia manipulation so far. There are studies showing correlation between the microglia activation and its membrane potential, but it has not been clearly investigated if changes in the membrane potential are the cause or the consequence of the microglia activation.

It has been especially challenging to specifically investigate the role of activated microglia in their native environment due to their interactions with other types of glial cells and neurons. However, studies have demonstrated that optogenetic astrocyte manipulation has successfully shown the possibilities of cell-type specific opsin expression with the transgenic mice and viral delivery of opsin, opening up the potential for many interesting future research avenues in optogenetic microglia manipulation.

Microglia play various critical roles in the CNS such as regulating synapse formation/elimination through BDNF release (Coull et al., 2005; Gottmann et al., 2009; Ferrini and De Koninck, 2013), and phagocytic activity induced by chloride influx (Averaimo et al., 2010), among others. Intracellular calcium increase in microglia is a common signaling pathway leading to expression of the proinflammatory genes including cytokines and chemokines such as TNF-α and ROS. As many previous studies in neurons and astrocytes have shown that optogenetic ChR2 stimulation increases intracellular calcium, it is now possible to investigate the optogenetically induced calcium increase and its effect on microglia.

In the near future, we anticipate that additional optogenetic manipulations utilizing different receptors/activators, such as opto-XR (Airan et al., 2009) or optoSTIM1 (Kyung et al., 2015), will be employed to delve deeper into the specific roles and underlying mechanisms of microglia in brain functions.

### Oligodendrocytes

Oligodendrocytes wrap tightly around axons (referred to as myelin sheaths) to facilitate the transmission of electrical signals to the terminal, thus providing structural and functional support to neural networks of the CNS. Myelin sheaths created by oligodendrocytes reduce ion leakage and cell membrane capacitance, leading to an increase in the impulse speed and faster signal propagation along insulated axons. Destruction of myelinating oligodendrocytes causes several types of nervous system dysfunction, including motor circuit deficits and delayed sensory processing (Tkachev et al., 2003; Takahashi et al., 2011; Philips et al., 2013). As with other types of glial cells, therapeutic strategies for protecting oligodendrocytes to support maintenance of a healthy myelin structure and reconstruction in response to injury are needed.

Despite the importance of oligodendrocytes as morphological and functional supports in the nervous system, only a few studies describing direct optogenetic manipulation of oligodendrocytes (Yamazaki et al., 2014, 2019). In these studies, selective ChR2 expression in oligodendrocytes was achieved using transgenic mouse models. They used the proteolipid protein (PLP) promoter to create tTA protein specifically in oligodendrocytes. Then, this tTA protein activated the tetO promoter, responsible for ChR2 expression, resulting in ChR2(C128S)-EYFP fusion protein production within oligodendrocytes. This approach ensured precise and targeted ChR2 expression in these cells. This emphasizes the significance of optogenetic approaches in comprehending neural circuit function and plasticity (Yamazaki et al., 2019). Oligodendrocyte depolarization, achieved through optogenetics, leads to significant increases in excitatory synaptic responses and enhances long-term potentiation (LTP) at specific synapses in the hippocampus. These results suggest that optogenetic manipulation of oligodendrocytes can finely tune synaptic activity and influence information transfer between different regions of the nervous system, highlighting the role of oligodendrocyte depolarization in modulating synaptic activity in a region- and cell type-specific manner.

Moreover, oligodendrocytes expressing ChR2 were applied to study the functional plasticity of white matter in the hippocampus (Yamazaki et al., 2014). Researchers observed that light-induced depolarization of oligodendrocytes led to both short-term and long-term facilitation of axonal conduction, depending on the magnitude of oligodendrocyte depolarization. These findings demonstrate that oligodendrocyte depolarization actively induces functional plastic changes in white matter. Therefore, optogenetic control of oligodendrocytes can significantly contribute to our understanding of how white matter responds to neural activity and how it influences brain functions.

As neurons lack sufficient spontaneous repair capacity, the control of the nervous system using oligodendrocytes is necessary to ensure functional recovery from damage to the CNS. In contrast to neurons, oligodendrocytes cannot generate action potentials; however, they respond to ion influx. In particular, the influx of calcium ions through the calcium channels of oligodendrocyte progenitor cells promotes their migration, differentiation, and myelination (Soliven, 2001; Cheli et al., 2015), indicating that the regulation of calcium ion fluctuations in oligodendrocytes may play a key role in nervous system maturation. Optogenetic manipulation of oligodendrocytes through the introduction of calcium-permeable ChR2 may enable precise control of the reciprocal interactions between neurons and glia.

### Schwann cells

The Schwann cell is a type of glial cell in the PNS, with equivalent functions to those of oligodendrocytes in the CNS. However, the structure of Schwann cells differs from that of oligodendrocytes. An individual Schwann cell can enclose only one axon, forming a myelin sheath, whereas a single oligodendrocyte forms myelin sheaths around segments of several adjacent axons. Studies on the functions of Schwann cells have extensively reported their pivotal role in increasing the conduction of electrical signals via myelination (Salzer, 2015). Additionally, they supply nutrients to neurons (Frostick et al., 1998; Boyd and Gordon, 2003) and modulate synaptic activity (Cao and Ko, 2007; Feng and Ko, 2008). Moreover, Schwann cells recognize damaged areas of the PNS and rapidly transform their phenotype into dedifferentiated and redifferentiated forms to assist in the regeneration of damaged neurons. Despite limitations regarding the functional recovery of regenerated nerves, peripheral nerves are more readily regenerated after injury than those of the CNS, due to the functions of Schwann cells in the PNS repair process.

**Figure 4:**
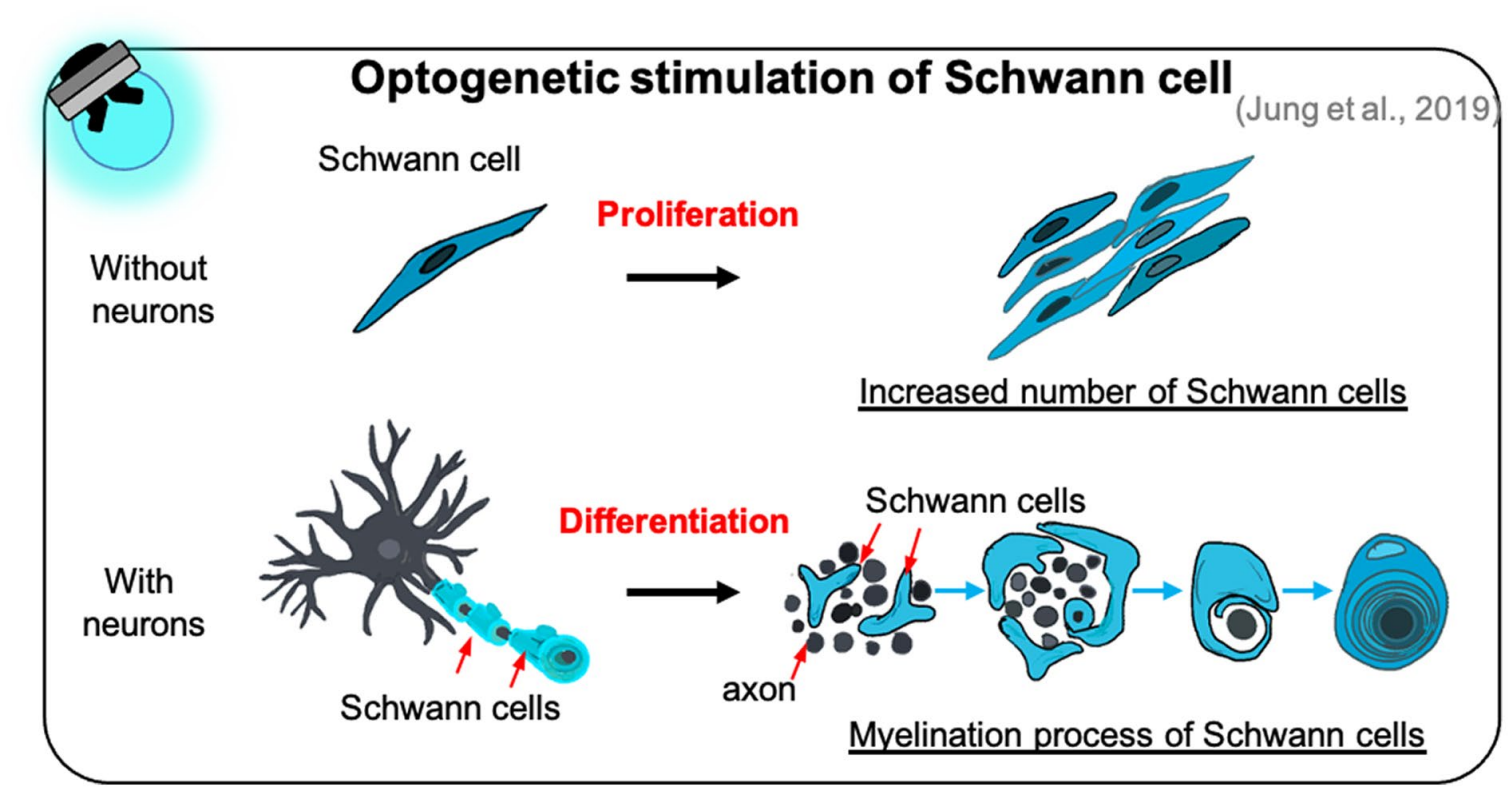
The effect of optogenetic Schwann cell manipulation on proliferation and differentiation. Optogenetic Schwann cells stimulate their proliferation in the Schwann cell monoculture without neuron (top) and differentiation to form myelin sheath in the Schwann cells-neuron coculture (bottom).

A few studies have reported that optogenetic manipulation of Schwann cells promotes their proliferation and differentiation, and accelerates the myelination of neurons cocultured with Schwann cells (Jung et al., 2019). Through LED irradiation of optogenetically modified Schwann cells, the number of proliferating Schwann cells increased substantially over time in the absence of neurons (Figure 4). Furthermore, optogenetically treated Schwann cells showed increased expression of myelin-related proteins, such as early growth response protein 2 and myelin basic protein, resulting in the formation of a compact myelin sheath in the presence of neurons (Figure 4). Surprisingly, these effects depended on intracellular calcium signaling in optogenetically modified Schwann cells; increased calcium levels were induced in these cells through T-type voltage-gated calcium channels and mobilization of inositol 1,4,5-trisphosphate-, caffeine-, and ryanodine-sensitive stores. Although controlling optogenetically modified Schwann cells is essential for optimizing their growth and function, the most effective method of manipulating optogenetic Schwann cell has not yet been determined. Moreover, there is a need for studies on the functions of optogenetically modified Schwann cells cocultured with other types of neurons and on their activities under *in vivo* conditions.

Recent research has demonstrated that PNS myelination is accelerated when optogenetic Schwann cells are used rather than optogenetic neurons (Jung et al., 2020). When LED irradiation was applied to optogenetically stimulated Schwann cells and neurons in a neuron-Schwann cell coculture model, the expression of several myelin-promoting factors increased more sharply in the Schwann cells, and the g-ratio (the ratio of the inner diameter to the total outer diameter) of myelinated axons in optogenetically stimulated Schwann cells was similar to the theoretical value for optimal fiber conduction. Similarly, optogenetic glial manipulation strongly promoted PNS myelin sheath maturation compared to the use of optogenetic neurons. These findings indicate that appropriate cell selection for optogenetic applications may improve our understanding of developmental and neurodegenerative diseases.

Furthermore, optogenetic techniques were applied to nociceptive Schwann cells, resulting in pain-like responses in experimental animals. This approach allowed precise manipulation of Schwann cell activity without directly stimulating sensory nerves (Abdo et al., 2019). This discovery highlights the intricate role of glial cells in sensory perception, particularly in sensing mechanical stimuli and contributing to pain modulation. The potential application of optogenetics introduced in controlling gene expression in Schwann cells for spinal cord regeneration (Muna, 2020). In this study, Schwann cells were optogenetically engineered to respond to near-infrared light (NIRW) stimulation, offering a non-invasive and precise approach to the production of therapeutic agents. These Schwann cells were stably engineered to express a NIRW light-responsive adenylyl cyclase called IlaM5. Upon exposure to NIRW irradiation, these engineered Schwann cells activated the expression of a reporter luciferase from a cAMP-dependent promoter, resulting in a several-fold increase in luciferase levels following 1 h of NIRW irradiation. Notably, the activation levels were even higher when biliverdin, the chromophore for IlaM5, was externally added to the Schwann cells. This innovative approach has significant implications for translational applications in spinal cord regeneration therapies, offering a promising avenue for precise and non-invasive control over gene expression in Schwann cells, which can support axon regeneration in cases of spinal cord injuries associated with permanent neurological deficits.

## Challenges and future considerations

Advances in optogenetic glial cell manipulation have opened up new perspectives on nervous system development. Moreover, these techniques hold excellent potential for enhancing nerve function and uncovering the mechanisms of neuron–glia communication. However, these approaches have only been applied to a limited number of glial cell types, and further studies are required to determine the molecular pathways through which each type of optogenetic glial cell regulates the physiology of the CNS and PNS.

It is important to note that achieving long-term and stable opsin expression in glial cells for clinical applications can be challenging, and there may be limitations to how long expression can be maintained. Achieving long-term and stable opsin expression in astrocytes for clinical applications presents challenges, with the duration of expression influenced by factors such as the viral vector, promoter, delivery method, and individual patient factors.

1. Viral vector selection: Utilizing a viral vector known for stable, long-term transgene expression is crucial. Adeno-associated viruses (AAV), renowned for their relatively enduring expression, hold paramount importance in astrocyte optogenetics for clinical use. Certain AAV serotypes have received regulatory approval for clinical gene therapy, facilitating potential clinical trials and novel therapy development.
2. Cell-specific promoters: The choice of a promoter directing opsin expression specifically in astrocytes ensures precise targeting. Cell type-specific promoters minimize off-target effects and unintended interactions between different cell types in the brain. Customizable to target specific astrocyte populations, these promoters tailor the therapy to the underlying mechanisms of neurological disorders.
3. Delivery method: Precision in targeting astrocytes while safeguarding neighboring cells is essential. Minimally invasive techniques prioritize patient safety, reduce tissue damage, and enhance accessibility and ease of administration. Real-time monitoring and control, ethical and regulatory compliance, translatability, and compatibility with combination therapies collectively underpin the development of safe and effective clinical astrocyte optogenetics.
4. Patient-specific considerations: Recognizing the variability in neurological disorders, treatment responses, and individual characteristics, personalized approaches are crucial. Tailoring glial cell optogenetic interventions allows for precise adjustments, prediction of long-term effects, and optimization of patient outcomes, advancing precision medicine in neuroscience and enhancing the quality of life for individuals affected by neurological disorders.

On the other hand, chemogenetic technologies, such as Designer Receptors Exclusively Activated by Designer Drugs (DREADDs), are widely employed by neuroscientists to identify the specific neural circuits and cellular signals that govern behaviors, perceptions, emotions, innate drives, and motor functions in various species from flies to nonhuman primates (Roth, 2016; Meister et al., 2021; Bossuyt et al., 2023). One key factor is that glial cells, including astrocytes and microglia, often communicate through G-protein-coupled receptors (GPCRs). The DREADD system, which relies on GPCRs engineered to respond to specific ligands, aligns well with the signaling pathways commonly found in glial cells. This makes it a more natural choice for modulating glial activity when compared to optogenetics. Additionally, chemogenetic manipulation can induce sustained changes in cellular activity as long as the ligand is present, allowing researchers to study longer-term effects. In contrast, optogenetic manipulation typically requires continuous light exposure, which may not be suitable for all experimental designs. Furthermore, chemogenetics is often preferred for *in vivo* studies because it can be more easily applied to freely moving animals. Researchers can administer the ligand systemically, allowing for precise control of glial activity in behaving animals. Moreover, chemogenetics, particularly when using systemically administered ligands, can penetrate deeply into tissues, making it suitable for manipulating glial cells in different brain regions. But, optogenetic stimulation may have limitations in terms of light penetration and tissue scattering. Lastly, the DREADD system offers flexibility in terms of temporal control. Researchers can administer the ligand at specific time points, allowing for controlled activation or inhibition of glial cells during particular phases of an experiment. DREADDs, while valuable, can have limitations. It exhibits poor reactivity with the endogenous ligand, potentially acting as dominant-negative forms of muscarinic receptors. This limitation can hinder their ability to effectively modulate cellular responses. Moreover, there are reported instances of toxic effects associated with long-term DREADD expression, although this may not be exclusive to glial cells (Goossens et al., 2021). Additionally, DREADDs, as engineered receptors, have the potential to interfere with endogenous signaling cascades, which could disrupt normal cellular processes. In contrast, optogenetic proteins, though exogenous, are less likely to disrupt endogenous signaling cascades. The choice between DREADDs and optogenetics should consider the specific research goals and potential drawbacks associated with each method.

Nevertheless, evidence supporting the importance of optogenetic glial manipulation is mounting. Given that damaged neurons cannot regenerate spontaneously and require structural and trophic support from glial cells, optogenetic strategies involving glial cells are reasonable for repair of damaged nerves. Ongoing research is aiming to determine (1) whether optogenetic manipulation of all types of glial cells can better control nerve function organization than optogenetic neuronal activation, and (2) the optimal expression level of the opsin gene to initiate the excitation of glial cells and maintain the activity of excitable cells. In addition, several challenges remain, including elucidating (1) whether optogenetic glial cells can be applied to correct nervous system dysfunction and (2) whether the effects of optogenetically activated cells and tissues are similar *in vitro* and *in vivo* and, ultimately, in the context of clinical application.

To apply optogenetics therapeutically in the clinical setting, it is important to focus on the delivery and safety of optogenetic tools. An *in-vitro* optogenetic study using human-derived cells provided critical evidence that the application of optogenetics to humans is possible (Zhuge et al., 2014; Klapper et al., 2017). According to high-quality review papers (Williams and Denison, 2013; Cheng et al., 2016; Shen et al., 2020), the therapeutic potential of optogenetic techniques appears to be substantial. Nevertheless, for clinical application of opsin gene activation in a specific cell type or brain region, invasive “gene conveyance” is required via viral injection into the target location. This injection leads to scar tissue formation and is susceptible to infection, which can cause immunogenic conditions. Additionally, a method for assessing the expression of the implanted opsin gene in human cells, and for mitigating damage caused by the temperature increase induced by the lights used for optogenetic stimulation, is still urgently needed.

## Author contributions

SH: conceptualization ideas, formulation or evolution of overarching research goals and aims. KJ, J-HP, and SH: writing (original draft) preparation, creation and/or presentation of the published work, specifically writing the initial draft. J-HP and SH: visualization, preparation, creation and/or presentation of the published work, specifically visualization/data presentation. All authors contributed to the article and approved the submitted version.
